# Analysis of affecting factors on patient safety culture in public and private hospitals in Iran

**DOI:** 10.1186/s12913-019-4863-x

**Published:** 2019-12-30

**Authors:** Amir Hossein Khoshakhlagh, Elham Khatooni, Isa Akbarzadeh, Saeid Yazdanirad, Ali Sheidaei

**Affiliations:** 1 Department of Occupational Health, School of public Health, Tehran University of Medical Sciences, Tehran, Iran; 2 Students’ Scientific Research Center, Tehran University of Medical Sciences, Tehran, Iran; 3 Department of Epidemiology and Biostatistics, School of Public Health, Tehran University of Medical Sciences, Tehran, Iran; 4 School of Health, Shahrekord University of Medical Sciences, Shahrekord, Iran; 5 Department of Biostatistics, Faculty of Paramedical Sciences, Shahid Beheshti University of Medical Sciences, Tehran, Iran

**Keywords:** Patient safety culture, Shift work, Job burnout, Path analysis

## Abstract

**Background:**

Patient safety culture is one of the main components of the quality of health services and is one of the main priorities of health studies. Accordingly, this study aimed to determine and compare the views of healthcare staff on the patient safety culture and the impact of effective factors on patient safety culture in public and private hospitals in Tehran, Iran.

**Methods:**

This cross-sectional study was carried out on a sample of 1203 health care workers employed in three public and three private hospitals in Tehran, Iran. Stratified random sampling was used in this study. Data were collected using the Maslach burnout inventory and patient safety culture questionnaire (HSOPSC). IBM SPSS v22 and Amos v23 were used to perform path analysis.

**Results:**

Eight hundred sixty-seven (72.57%) females and 747 (27.43%) males with a mean age of 33.88 ± 7.66 were included. The average percentage of positive responses to the safety culture questionnaire in public and private hospitals was 65.5 and 58.3%, respectively. The strengths of patient safety culture in public hospitals were in three dimensions including non-punitive response to errors (80%), organizational learning—continuous improvement (79.77%), and overall perceptions of patient safety (75.16%), and in private hospitals, were three dimensions including non-punitive responses to errors (71.41%), organizational learning & continuous improvement (69.24%), and teamwork within units (62.35%). The type of hospital and work-shift hours influenced the burnout and patient safety questionnaire scores (*P*-value < 0.05). The path analysis results indicate the fitness of the proposed model (RMSEA = 0.024). The results showed a negative impact of a work shift (β = − 0.791), occupational burnout (β = − 0.554) and hospital type (β = − 0.147) on the observance of patient safety culture.

**Conclusion:**

Providing feedback on errors and requirements for the frequent incident reporting, and patient information exchange seem necessary to promote the patient safety culture. Also, considering the negative impact of the shift work and burnout on patient safety culture, by planning and managing these factors appropriately, correct actions could be designed to improve the safety culture.

## Background

Patient safety culture is one of the essential components for providing quality healthcare services. The importance of patient safety culture has led to numerous studies in this regard in various health centers, including hospitals [1]. Medical errors are one of the five common causes of death worldwide [2].

The world health organization has estimated that tens of millions of patients are the victims of injuries and deaths from unprotected medical care and activities around the world [1]. For example, medical errors in the United States annually result in 44,000 to 98,000 deaths in hospitals. Based on the available evidence, it is estimated that in developed countries, 1 out of 10 patients, will be injured during services [1–4].

The differences between the private and public healthcare sectors are in regulation, payments, and training. Private and public healthcare sectors have their strengths and weaknesses and do not have a lot in common in the context of the working environment. Working as a nurse or physician do not differ in either sector, but still, some points may affect their decision to work in either sector. For instance, working in the public sector will be a busy and crowded job. Government regulations and training will also usually be accomplished more rigorously in the public sector when in private hospitals, salaries are more attractive [5, 6].

Despite all of the efforts made by healthcare organizations, the prevalence of medical errors is still high [2]. This high rate can be due to cultural factors and lack safety culture in healthcare workers [7]. The most crucial obstacle to improve patient care safety is the safety culture of health care organizations [8]. A rectifier safety culture is vital for improving patient safety [7, 8]. Patient safety culture is a subset of organizational culture and is defined as a set of values, attitudes, perceptions, beliefs, and behaviors that support the safe conduct of individuals’ activities in health organizations [7, 9]. The critical components of the patient safety culture include a common belief that the risk of responsibility for health care is high, organizational commitment to detect and analyze errors and injuries to the patient, and ultimately creating an environment that balances the need for error reporting and the need for disciplinary action [7–9].

A positive safety culture directs healthcare providers’ behaviors, so that patient safety becomes one of their highest priorities; this includes elements such as organizational learning, teamwork, open communications, feedback and non-punitive responses to errors, and shared cultural perceptions based on the importance of safety [1, 8]. A positive safety culture can encourage health providers to report and analyze their errors, which is an effective tool for improving safety because the first step toward creating a positive safety culture is to assess the current safety culture [1, 4]. On the other hand, hospitals should create a patient safety culture among their employees before implementing structural interventions; therefore, the importance of knowing the existing culture of patient safety should be emphasized [4, 8]. An assessment of the organization safety culture makes it possible to obtain a clear overview of the patient safety aspects that require more attention. It also allows hospitals to identify the strengths and weaknesses of their safety culture and patient safety issues and also compare their patient safety culture score with other hospitals [1, 4, 8].

Occupational burnout is a product of long-term stress in the workplace [10]. Symptoms of this syndrome are manifested when an individual’s skills are not enough to meet the needs of the workplace [11]. Emotional exhaustion (chronic fatigue, sleep disturbances, various physical symptoms) as a decrease in energy and feelings of depletion of mental capacity, depersonalization (negative and feeling less reactions, with excessive disregard for co-workers and clients, feeling guilty, isolation, decreased work and daily activities) means a person’s mental separation from his or her job and a decrease in his/ her personal accomplishment (reduced sense of competence and success in the profession, dissatisfaction with work, feelings of failure and disability, loss of ability to understand and perceive, the persistent sense of abuse and exploitation, and reduction in job performance) and are three different dimensions of burnout [10, 11]. Medical staff (physician, nurse, nursing assistant) due to exposure to stress such as patients mortality, interpersonal problems, high workload, low social support, exposure to a large number of patients per day, emergency decision making based on inadequate information and being responsible for results of these decisions, efforts alongside stress to avoid any mistakes, exposure to violence and threats at work and work shifts are more likely to be involved in this syndrome than other occupations [10, 12].

Examining the patient safety in hospitals is more important for reasons such as job burnout, occupational stress, and psychological load and higher levels of stress [12, 13]. On the other hand, the lack of a study that explores different factors such as job burnout and individual and organizational factors on patient safety culture in both public and private hospitals reinforces the importance of studying in this regard. Therefore, considering the importance of this issue, the present study aimed to investigate the effect of demographic factors and job burn out on the patient safety culture, using path analysis in public and private hospitals in Iran. The hypotheses of the study were whether the patient safety culture in private hospitals is better than public hospitals (first hypothesis), the patient safety culture is lower in shift workers (second hypothesis), and occupational burnout has a direct effect on patient safety culture (third hypothesis).

## Methods

### Study design, setting, and sample

This cross-sectional study was conducted in selected public and private hospitals in Tehran, Iran (three public hospitals, and three private hospitals). The investigation was carried out from September 2017 to August 2018 in this study, full-time employment in the hospital, the physical and mental desire and ability to participate, and having more than 6 months of work in the hospital were defined as inclusion criteria.

Recruited participants were chosen by stratified sampling method proportional to the size of the hospital units. A list of hospital departments was first prepared, and then according to the percentage of staff in each department, questionnaires were randomly distributed among the staff working in different parts of each hospital.

### Measurement tools

#### Demographic questionnaire

In this questionnaire, demographic information including age, sex, work experience, marital status, work unit, and shift work were collected.

#### Maslach burnout inventory

The Maslach burnout inventory includes 22 items that measure three aspects of job burnout (emotional exhaustion, personal accomplishment, and depersonalization). In 1981, Maslach et al. measured the internal reliability coefficient for emotional exhaustion as 0.9, depersonalization as 0.79, and personal accomplishment as 0.71 [14]. The ICC between 0.75 and 0.9 is considered as “good,” and more than 0.9 as “excellent.” To ensure that all dimensions (especially personal accomplishment, which its ICC is below 0.75) are in the “good” range, we calculated the ICC within our data. The reliability of the Persian version of the questionnaire was obtained 0.87 [15].

The questions (1, 2, 3, 6, 8, 13, 14, 16, and 20) are related to the emotional exhaustion subscale. Questions (5, 10, 11, 15, and 22) also relate to the emotional exhaustion subscale, as well as questions (4, 7, 9, 12, 17, 18, 19, and 21) are related to the personal accomplishment. A Likert scale was used. The scoring options for this test were as “never” with score of 0, “very low” with score of 1, “low” with score of 2, “average” with score of 3, “medium to high” with score of 4, “high” with score of 5, and “very high” with score of 6. However, the questions (1, 2, 3, 5, 6, 8, 10, 11, 13, 14, 15, 16, 20, and 22) of this questionnaire are rated inversely and the questions (4, 7, 9, 12, 17, 18, 19, and 21) are rated directly [14, 15].

#### Patient safety questionnaire

HSOPSC is a self-report questionnaire with 12 dimensions. The validity and reliability of this questionnaire were confirmed in the study by Chen et al. in 2010 [16]. Moghri et al. have translated this questionnaire into Persian and reviewed the translation validity. The reliability of this questionnaire was between 0.57 and 0.8 [17]. As several ICCs for this questionnaire are less than 0.6, the based ICCs in our data are presented in Table 3.

A Likert scale was used. Scores 1 and 2 were expressed contrary to patient safety, 3 was neutral, and 4 and 5 were positive. In order to calculate the hospital’s score on a safety culture dimension, the average percent positive answers on all questions in the dimension was obtained. In order to acquire percent positive scores, negatively worded items were reversed [16].

#### Sample size

**Fig. 1:**
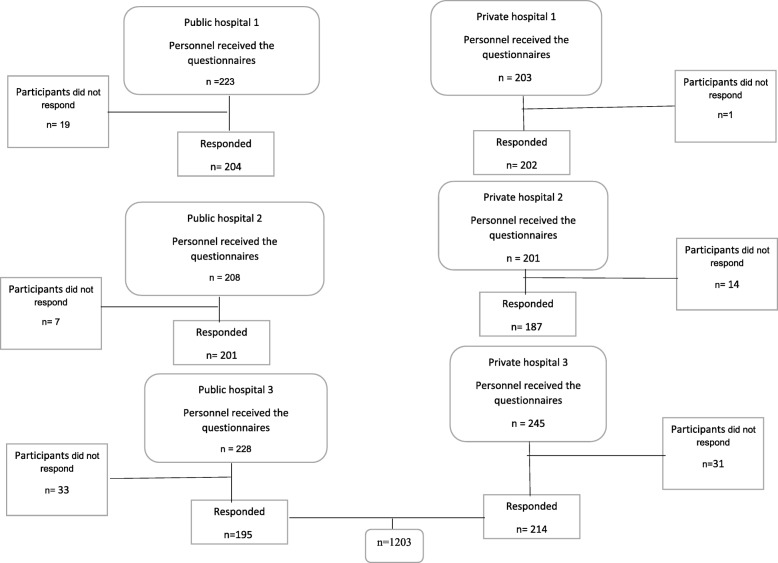
Participants in the study

It is advised that for path analysis models, the best sample size should be 20 times fold the number of parameters in path analysis [18]. There are two questionnaires with 12 and 3 dimensions in this study. A mean parameter and a variance parameter should be estimated for each dimension. Besides, our primary goal was comparing between public and private hospitals. Therefore, we stratified our data on the type of hospital with two levels. Considering 30 parameters to estimate in 2 strata, we needed 1200 sample size (30*2*20) (Fig. 1).

### Statistical analysis of data

Data were analyzed using IBM SPSS v22 and AMOS v23. Missing answers are excluded when presenting percentages of answers to the study items. Descriptive statistics (mean and standard deviation) of the demographic characteristics of participants, characteristics of hospitals, and the average percentage of positive answers on patient safety culture were calculated. In order to assess the normality of the data, the Kolmogorov-Smirnov test was conducted. Next, inferential statistics, including Pearson correlation for exploring the association between dimensions and questionnaire scores, one-way ANOVA for comparing the difference of scores between a categorical variable with more than two categories, and independent sample t-test for comparing scores between 2 categories of binary variables were used. Therefore, independent T-test was used to compare the mean scores of each dimension of the questionnaire at the levels of gender and type of hospital. One-way analysis of variance was used to compare the meanings at levels of other variables. Finally, a path analysis was done to assess the relationship between covariates and outcomes. In this study, the RMSEA index was less than 0.05, the CMIN/DF index was less than 2, and the GFI index was higher than 0.9 as the indicators of the fitness of the path analysis model [19].

## Results

In this study, 1203 subjects were entered. Table 1 shows the demographic characteristics of the subjects divided by different variables. From the total number of 1203 cases, 867 people (72.57%) were female. Also, most participants in the study, 689 people (57.27%) were nurses. The age group of 30–39 years old with 504 people (41.9%) had the highest percentage among the age groups. Most of the medical staff in the hospitals were with contractual employment status (66.5%), and finally, because of the stratified sampling method used in this study, there was no difference between the number of the subjects taken from public and private hospitals (the study includes three private hospitals and three public hospitals).

**Table 1:** Demographic characteristics of the subjects (*n* = 1203)

Variables	Number	Percent (%)
Age group		
Under 30	407	33.83
30–39	504	41.9
40–49	257	21.36
≥ 50	35	2.91
Gender		
Male	336	27.93
Female	867	72.07
Work experience		
Under 5 years	898	74.65
6–10 years	148	12.3
11–15 years	114	9.48
More than 15 years	43	3.57
Unit		
Medicine (non-surgical)	207	17.21
Surgery	117	9.73
Women	72	5.99
Children	33	2.74
Psychiatry	20	1.66
Intensive Care Unit	556	46.22
Emergency	128	10.64
Laboratory	48	3.98
Occupational medicine	16	1.33
Operating room	6	0.5
Type of employment		
Permanent	168	13.97
Temporary to permanent	90	7.48
Contractual	800	66.5
Conscription law’s conscripts	62	5.15
Others	83	6.9
Type of hospital		
Public	600	49.88
Private	603	50.12
Shift work		
Yes	819	68.08
No	384	31.92

The highest score among dimensions of the questionnaire was related to the personal accomplishment rate, with an average of 3.94 and a standard deviation of 1.82. On the other hand, the lowest score is for depersonalization dimensions, with an average of 2.49 and a standard deviation of 1.71.

In the public hospitals, the average of personal accomplishment feeling score (4.19) was significantly higher than the average scores of this dimension in private hospitals (3.69) (*P*-value < 0.001). Meanwhile, in the depersonalization dimension, the average scores in public hospitals are 2.3, and it is 2.67 in private hospitals (*P*-value< 0.001). For the emotional exhaustion, the mean scores of public and private hospitals are 2.33 and 2.68, respectively (*P*-value < 0.001). In the shift-work cases, the average total score of job burnout was 2.77 in public and was 3.48 in private hospitals (*P*-value< 0.001). All values and comparisons are available in Table 7 in Appendix. There was no significant difference between occupational burnout score and age group, work experience, sex, and place of service (*P*-value> 0.05), but this difference was significant in hospital type and shift work (*P*-value < 0.05) (Table 7 in Appendix).

Here, as well as the burnout questionnaire, the demographic variables of the type of hospital and the shift work had a significant effect on the mean scores of the dimensions of the patient safety culture questionnaire. The highest score was for the communication openness dimension, which has 5.93 mean and 1.33 standard deviation. Also, the lowest score is for the non-punitive response to errors with a mean of 3.35 and a standard deviation of 1.19.

There was no significant difference between the patient safety culture score and the age group, work experience, sex, and unit of service (*P*-value> 0.05). However, this difference was significant in the type of hospital and shift work (*P*-value < 0.05) (Table 8 in Appendix).

In this study, public hospitals healthcare personnel gave the following scores about the safety culture of their work units: 24% scored for high, 39% scored for very good, 13% scored for acceptable, 13% scored for poor, 11% scored for failed; and in private hospitals: 18% scored for high, 30% scored for very good, 15% scored for acceptable, 19% scored for poor, 18% scored for failed.

The average percentage of positive responses to the safety culture questionnaire in public and private hospitals was 65.5 and 58.3%, respectively. The results of the assessment of the studied population views on the 12 dimensions of patient safety by the public and private hospitals are presented in Table 2. From the viewpoint of participants in public hospitals, three dimensions of safety culture including non-punitive responses to errors and mistakes (80%), organizational learning & continuous improvement (79.77%), overall perceptions of patient safety (75.16%); and in the private hospitals three dimensions of non-punitive responses to errors and mistakes (71.41%), organizational learning & continuous improvement (69.24%) and teamwork within units (62.35%) were identified as the strengths of the safety culture. The dimensions of supervisor/manager expectations & actions promoting patient safety (42.18% in public hospitals and 34.67% in private hospitals), feedback & communication about error (28.81% in public hospitals and 32.24% in private hospitals) and the frequency of events reported (32.48% in public hospitals and 47.41% in private hospitals) were identified as three dimensions requiring improvement from the viewpoint of the studied population.

**Table 2:** The average percentage of positive responses to the 12 dimensions of patient safety culture from the viewpoint of the studied population (*n* = 1203)

Dimensions of patient safety culture	The average percentage of positive responses		*P* value
	Public hospitals	Private hospitals	
Teamwork within units	68.66	57.37	0.08
Supervisor/manager expectations & actions promoting patient safety	42.18	34.67	0.31
Organizational learning & continuous improvement	79.77	69.24	0.07
Management support for patient safety	66.66	59.09	0.24
Overall perceptions of patient safety	75.16	52.54	0.001
Feedback & communication about error	28.81	23.42	0.33
Communication openness	72.44	59.75	0.07
Frequency of events reported	23.48	47.41	< 0.001
Teamwork across units	75	62.35	0.05
Staffing	72.33	61.02	0.10
Handoffs & transitions	64.73	52.85	0.08
Non-punitive Response to Errors	80	71.41	0.14

Internal correlations of questions in different dimensions of two questionnaires are presented in Table 3. As the results show, all the values for the Maslach burnout inventory questionnaire are higher than 0.8, and the reliability of them is confirmed. The lowest internal consistency in the patient safety culture questionnaire was related to the area of staffing (0.62), and the most were related to the frequency of events reported (0.81). All the values are in the acceptable range (near 0.75) and are higher than previous studies [17].

**Table 3:** Internal Correlations in Different Dimensions of Two Questionnaires

Dimensions	Number of questions	Internal Correlations
Teamwork within units	4	0.73
Supervisor/manager expectations & actions promoting patient safety	4	0.8
Organizational learning & continuous improvement	3	0.73
Management support for patient safety	3	0.8
Overall perceptions of patient safety	4	0.73
Feedback & communication about error	3	0.75
Communication openness	3	0.7
Frequency of events reported	3	0.84
Teamwork across units	4	0.78
Staffing	4	0.62
Handoffs & transitions	4	0.78
Non-punitive Response to Errors	3	0.77
Emotional exhaustion	9	0.81
Depersonalization	5	0.83
Personal accomplishment	8	0.87

In Table 4, the fitness indices of the path analysis model are presented.

**Table 4:** Fitness indices of the model

Indices	Statistics	Fitness	Obtained values
Absolute fitness indices	Goodness-of-fit index (GFI)	> 0.9	0.999
	Adjusted goodness-of-fit index (AGFI)	> 0.9	0.993
Comparative fitness indices	Normed fit index (NFI)	> 0.9	0.999
	Comparative fit index (CFI)	> 0.9	1
	Incremental fit index (IFI)	> 0.9	1
Normed fit index	Parsimonious normed fit index (PNFI)	> 0.5	0.167
	Root mean squared error of approximation (RMSEA)	< 0.05	0.024
	Normed Chi-square (CMIN/DF)	< 2	1.701

**Fig. 2:**
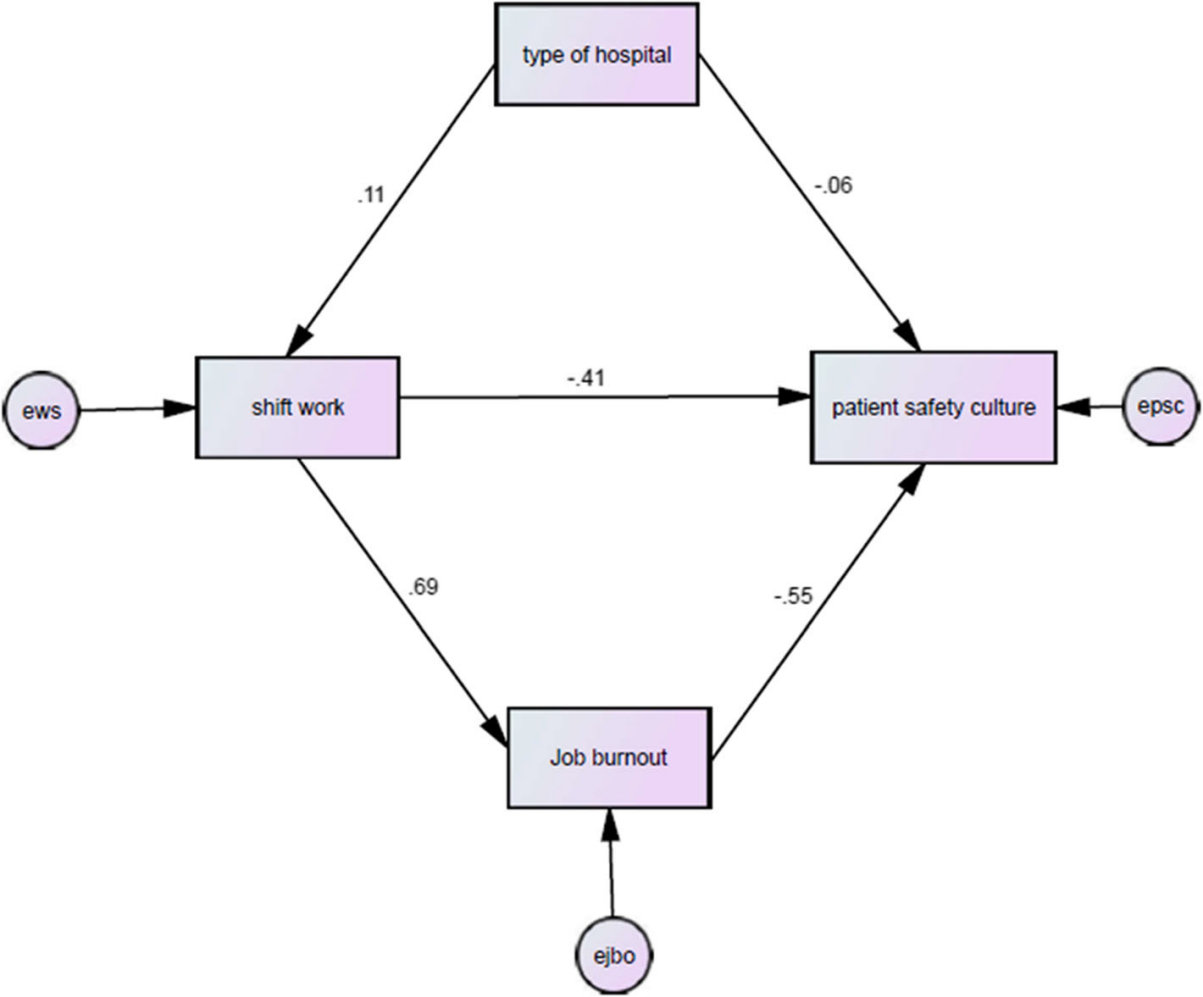
Theoretical model of the current study

In Fig. 2 and Table 5, the coefficients of the path of the study variables are shown.

**Table 5:** standard path coefficients of path analysis model of effecting variables on the patient safety culture among the studied population (*n* = 1203)

Role of the variable	standard path coefficients	Standard error	*P*-value
Independent→ dependent			
Hospital type → shift work	0.106	0.027	< 0.001
Shift work → occupational burnout	0.690	0.021	< 0.001
Shift work → patient safety culture	−0.409	0.046	< 0.001
Hospital type → patient safety culture	−0.063	0.032	< 0.001
Occupational burnout → patient safety culture	−0.554	0.044	< 0.001

Given that the RMSEA index is less than 5% and the CMIN/DF index is less than two, and the GFI index is more than 0.9, the goodness of the proposed model in the path analysis can be ensured. Also, the results show the significance of the coefficients presented in the path analysis. Table 6 shows the direct and indirect effects of the variables in the study on the patient safety culture. The results of this table show the negative impact of a work shift (β = − 0.791), occupational burnout (β = − 0.554) and hospital type (β = − 0.147) on the observance of patient safety culture.

**Table 6:** Direct, indirect, and total effects of variables on the patient’s safety culture among the study population (*n* = 1203)

Variables	Direct effect	Indirect effect	Total effect
Shift work	−0.409	−0.382	− 0.791
Type of hospital	−0.063	− 0.084	−0.147
Occupational burnout	−0.554	0	−0.554

## Discussion

This research is the first comprehensive study to examine the relationship between factors affecting the patient safety culture in private and public hospitals with different healthcare workers in Iran.

In this study, two high standard tools were used to evaluate patient safety culture and occupational burnout.

Medical personnel as a subset of a human society who are involved with occupational burnout and stress and high occupational burden in comparison with other groups of the population are more exposed to physical and emotional problems than the ordinary society. This issue is highly important since the problems of this group directly affect the erosion of medical errors, and consequently, the health of people in the community; it has a double impact. Reducing medical errors is not possible by directly increasing the observance of the safety culture by medical personnel. Therefore, this study aimed to investigate the burnout and demographic factors affecting the level of patient safety culture. The results of this study showed a negative effect of burnout on patient safety culture. There was also a significant difference in patient safety culture in different types of hospitals, in which the patient safety culture was better in public than in private hospitals.

Today, in order to provide a desirable and standard healthcare service, a notable emphasis is placed on the physical and mental health of healthcare personnel. Occupational burnout is a very crucial topic because of the dramatic changes that bring to personal, family, and general and professional health [20].

Demographic factors, including age group, gender, work experience, work unit, and type of employment, do not significantly affect the level of patient safety culture. This result is precious because these variables are commonly only altered in a system by staff turnover [21].

The first hypothesis, which was the better patient safety culture in private hospitals, was rejected according to the results of the study. The patient safety culture score in public hospitals was higher than private ones, which indicates that health care in public hospitals is better than private hospitals. This could be the result of a more excellent investment of the Ministry of Health in its hospitals compared with private hospitals regarding patient service quality and safety. Results of the present study are consistent with the results of these studies [20, 22].

The results confirmed the second hypothesis, which stated that the patient safety culture is at lower levels in shift workers. The patient safety culture score for shift workers was lower than those who were not, which means shift working has a negative impact on the safety culture of the patient. In this regard, the negative impact of shift work on the reduction of observance of the patient safety culture can be explained by the double fatigue resulting from shift work [23]. In a study, results showed that shift work and night work would reduce the quality of the patient safety and, consequently, increase medical errors [24].

Based on the results of the 12 dimensions of patient safety culture, two dimensions of organizational learning & continuous improvement, and non-punitive responses to errors and mistakes were identified as the strengths of safety culture in public and private hospitals. Additionally, the overall perceptions of patient safety in public hospitals and the teamwork within hospital units have also been recognized as a strong point in the safety culture in private hospitals.

In a study conducted in 68 hospitals in Lebanon, as in this study, organizational learning & continuous improvement was the highest score, but unlike the results of the present study, the non-punitive response to errors and mistakes had the lowest score [25].

In a survey, in which the patient safety culture was evaluated in the emergency departments of 33 non-academic hospitals in the Netherlands, the subjects chose teamwork within the emergency department and open communications as the best patient safety dimensions [26].

In another study, organizational learning & continuous improvement and feedback & communication about error were indicated as strengths and frequency of events reported, non-punitive responses to errors and mistakes, staffing, and teamwork within units were reported as weaknesses [22].

Based on the results of this study, the dimensions required for improvement in both public and private hospitals were three dimensions of supervisor/manager expectations & actions promoting patient safety, feedback & communication about errors and the frequency of unwanted errors reported.

In a survey, results showed that dimensions with the lowest score were the frequency of events reported, teamwork between units, and management support [26].

In another study, the dimensions with the lowest score were handoffs & transitions of patient information between the department and the shift, the staffing, and the non-punitive response to errors and mistakes [25].

In the context of the weakness of management support for patient safety, in terms of manager’s expectations and actions to improve patient safety, it is worth noting that the promotion of the hospital’s safety culture is a major development and requires the change in the values, beliefs, and behavior of the organization staff in line with the values of the safety culture; and such a change requires the support of senior executives, leaders, and supervisors [25, 26].

Given the fact that the hospitals are weak in terms of feedback and informing others about the errors and frequencies of unwanted errors reporting, they will not have the opportunity to take lessons and to improve safety culture from errors and mistakes by exploring the reasons of these errors and the way of handling them [26, 27].

In this regard, the establishment of a systematic and comprehensive system for reporting errors and incidents seems vital because it leads to the identification of types, the nature, and cause of errors, and design processes and adopts measures to reduce or eliminate similar errors and occurrences, which diffidently will be very effective [27].

In this study, 24% of healthcare personnel of public hospitals gave as perfect score, 39% as very good, 13% as acceptable, 13% as poor and finally 11% as failed to the safety of their working unit and in private hospitals 18% scored for a perfect score, 30% for very good, 15% for acceptable, 19% for poor, 18% for failed. This finding suggests that the development of different dimensions of safety culture in hospitals, especially private ones, needs improvement, and confirms these studies [7, 16]. In a study, 60% of the subjects rated hospital safety as excellent and very good, 33% acceptable, and 7% poor [22].

The results of the study are consistent with the third hypothesis, which indicates that burnout has a direct negative effect on the patient safety culture so that when the burnout increases, the patient safety culture decreases. The results of this study are consistent with the results of these studies [28, 29].

In other words, it can be claimed that since in this study burnout is known as an independent variable affecting the observance of patient safety culture, planning to reduce burnout and consequently increasing the observance of the patient safety culture that follows reducing hospital accidents can be considered as a concern for managers.

Occupational burnout score was lower among public hospitals healthcare staff than private hospitals. One reason for this can be that the goal in private hospitals is to reduce costs in order to increase profits and benefits, leading to a reduction in workforce and an increase in workload, which will lead to an increase in the burnout of healthcare personnel. The results of this study are consistent with the results of the study conducted in Sweden [30].

Burnout score was higher among shift workers than non-shift workers. In concluding this result of the study, it can be said that constant changing in sleep and awakening cycles causes psychological stress and family and personal problems. Longer working hours, heavier responsibilities, and lower family and social support, cause the amount of burnout to be higher. The results of this study are consistent with the study done in Thailand [31].

Existence of high levels of emotional exhaustion and depersonalization and lack of sense of personal accomplishment can trigger an alarm for the managers because, in the absence of appropriate planning to control it, it can lead to extensive damage to the health system [31].

Generally speaking, when people work in areas where there is lack of proper encouragement, induced sense of effectiveness, and insight, and the tasks are not well understood, the duties and policies are not well explained, the new and diverse approaches do not come up, the work environment is not pleasant and desirable, the situation does not have the conditions for mental comfort, people not only get occupational burnout but also they lose their attitudes toward the patient care.

### Limitations

This survey was a cross-sectional study, and causation cannot be investigated. The number of participants in the study was more in comparison to the existing studies, so the extended research team was needed. The results of this survey may not be generalized for other countries because of different patient safety culture structure.

## Conclusion

According to the results, it is possible to plan and manage shift work and burnout in order to improve the safety culture. Private hospitals should also pay more attention to the patient safety culture and focus their investments on improving the patient safety culture.

Also, based on the results, there is a need to pay more attention to improving the patient safety culture in the areas of supervisor/manager expectations & actions promoting patient safety, feedback and inform others about the errors and the frequency of unwanted incident reporting. The committed leadership of the organization in providing safe health care is recognized as one of the main factors behind patient safety improvement. Managers and supervisors of the organization are as leaders and should consider the system issues that exist within the organization to provide organizational and individual learning opportunities. Effective communication within the organization and providing feedback on error reporting will lead to organizational learning of errors and identify ways to prevent these errors in the future. Implementation of interventions to promote the patient safety culture in the studied hospitals and the evaluation of the impact of interventions is recommended for further research.

## Data Availability

The datasets used in the present study are accessible from the corresponding author on reasonable request.

## Appendix

**Table 7:** Demographic characteristics of the study population based on the Maslach burnout questionnaire (*n* = 1203)

Level	Maslach burnout questionnaire			
	Total	PA	D	EE
Total				
Mean	3.02	3.94	2.49	2.50
SD	0.49	1.82	1.71	1.74
Age groups				
Under 30				
Mean	3.02	3.98	2.47	2.47
SD	0.50	1.78	1.70	1.75
30–39				
Mean	3.02	3.97	2.46	2.48
SD	0.47	1.80	1.68	1.68
40–49				
Mean	3.04	3.83	2.56	2.61
SD	0.50	1.89	1.79	1.80
≥ 50				
Mean	3.01	3.85	2.55	2.51
SD	0.55	1.91	1.78	1.92
*P* value	0.92	0.71	0.87	0.75
Work experience				
Under 5				
Mean	3.02	3.90	2.51	2.52
SD	0.50	1.82	1.73	1.76
6–10				
Mean	3.01	4.09	2.42	2.39
SD	0.44	1.83	1.63	1.62
11–15				
Mean	3.05	3.97	2.46	2.55
SD	0.48	1.80	1.70	1.68
More than 15				
Mean	2.96	4.16	2.25	2.30
SD	0.45	1.82	1.72	1.67
*P* value	0.81	0.57	0.73	0.68
Hospital type				
Public				
Mean	3	4.19	2.30	2.33
SD	0.47	1.65	1.58	1.62
Private				
Mean	3.4	3.69	2.67	2.68
SD	0.51	1.94	1.81	1.83
*P* value	0.11	< 0.001*	< 0.001*	< 0.001*
Sex				
Female				
Mean	3.02	3.99	2.45	2.46
SD	0.48	1.80	1.69	1.69
Male				
Mean	3.04	3.81	2.57	2.62
SD	0.52	1.85	1.76	1.84
*P* value	0.43	0.11	0.28	0.15
Shift Work				
Yes				
Mean	3.48	2	4.29	4.35
SD	0.43	1.59	1.46	1.48
No				
Mean	2.77	4.99	1.51	1.51
SD	0.31	0.76	0.8	0.79
*P* value	< 0.001*	< 0.001*	< 0.001*	< 0.001*
Work unit				
Non-surgical				
Mean	2.96	4.13	2.29	2.30
SD	0.46	1.74	1.62	1.64
Surgery				
Mean	3.03	3.79	2.58	2.59
SD	0.50	1.88	1.78	1.81
Midwifery				
Mean	2.94	3.99	2.36	2.31
SD	0.49	1.79	1.75	1.73
Pediatrics				
Mean	3.06	3.89	2.51	2.62
SD	0.39	2.02	1.77	1.58
Psychiatry				
Mean	2.89	4.53	1.96	1.94
SD	0.37	1.51	1.30	1.35
intensive care				
Mean	3.05	3.84	2.58	2.60
SD	0.51	1.84	1.74	1.79
Emergency				
Mean	3.03	3.99	2.49	2.48
SD	0.51	1.79	1.72	1.75
Lab				
Mean	3.10	4.28	2.40	2.44
SD	0.46	1.60	1.50	1.58
Occupational medicine				
Mean	3.06	4.08	2.49	2.48
SD	0.40	1.91	1.65	1.63
Operating room				
Mean	3.12	3.71	2.83	2.76
SD	0.35	2.16	1.92	1.70
*P* value	0.38	0.45	0.58	0.63

* statistically significant result (*p* < 0.05)

**Table 8:** Demographic characteristics of the study population based on patient safety assessment questionnaire (*n* = 1203)

Level	Patient safety quality assessment questionnaire												
	Total	NRtE	HaT	S	TAU	FoER	CO	FaCAE	OPoPS	MSfPS	OLCI	SMEAPPS	TWU
Total													
mean	3.48	3.35	3.53	3.42	3.50	3.56	3.59	3.52	3.52	3.55	3.44	3.46	3.38
SD	1.22	1.19	1.34	1.14	1.35	1.34	1.33	1.41	1.29	1.34	1.19	1.26	1.04
Age groups													
Under 30													
mean	3.49	3.33	3.54	3.44	3.51	3.60	3.59	3.52	3.54	3.55	3.40	3.45	3.39
SD	1.21	1.17	1.32	1.14	1.34	1.35	1.33	1.38	1.30	1.34	1.20	1.27	1.03
30–39													
mean	3.51	3.39	3.56	3.44	3.52	3.58	3.62	3.55	3.53	3.58	3.46	3.51	3.40
SD	1.20	1.19	1.33	1.13	1.32	1.32	1.33	1.40	1.29	1.32	1.17	1.24	1.04
40–49													
mean	3.42	3.30	3.44	3.33	3.42	3.46	3.52	3.47	3.48	3.46	3.47	3.38	3.33
SD	1.25	1.23	1.40	1.16	1.42	1.35	1.31	1.47	1.30	1.39	1.21	1.27	1.07
Above 50													
mean	3.49	3.44	3.57	3.44	3.48	3.44	3.59	3.52	3.52	3.58	3.50	3.45	3.39
SD	1.24	1.23	1.31	1.25	1.36	1.34	1.37	1.47	1.31	1.17	1.25	1.27	1.06
*P* value	0.81	0.73	0.73	0.6	0.79	0.52	0.85	0.92	0.93	0.69	0.84	0.64	0.84
Work experience													
Under 5													
mean	3.46	3.33	3.50	3.40	3.48	3.52	3.55	3.49	3.49	3.52	3.41	3.44	3.36
SD	1.22	1.20	1.35	1.15	1.36	1.35	1.33	1.42	1.31	1.35	1.21	1.27	1.04
6–10													
mean	3.57	3.43	3.61	3.48	3.55	3.69	3.73	3.64	3.62	3.60	3.61	3.52	3.43
SD	1.20	1.20	1.32	1.13	1.32	1.33	1.30	1.39	1.24	1.31	1.14	1.22	1.04
11–15													
mean	3.51	3.38	3.55	3.41	3.54	3.61	3.63	3.57	3.55	3.60	3.42	3.50	3.42
SD	1.20	1.16	1.30	1.11	1.33	1.29	1.35	1.37	1.28	1.32	1.15	1.26	1.07
More than 15													
mean	3.63	3.46	3.69	3.51	3.62	3.69	3.71	3.68	3.70	3.71	3.66	3.55	3.59
SD	1.17	1.22	1.24	1.11	1.32	1.29	1.22	1.29	1.23	1.29	1.10	1.20	1.06
*P* value	0.6	0.76	0.66	0.85	0.84	0.42	0.4	0.5	0.56	0.71	0.17	0.8	0.46
Hospital type													
Public													
mean	3.64	3.48	3.69	3.54	3.67	3.77	3.77	3.71	3.70	3.70	3.59	3.61	3.53
SD	1.13	1.11	1.23	1.07	1.26	1.24	1.25	1.31	1.22	1.23	1.11	1.17	0.96
Private													
mean	3.32	3.23	3.37	3.29	3.33	3.34	3.41	3.33	3.35	3.39	3.30	3.30	3.24
SD	1.28	1.25	1.42	1.20	1.41	1.39	1.38	1.48	1.34	1.43	1.25	1.32	1.10
*P* value	< 0.001*	< 0.001*	< 0.001*	< 0.001*	< 0.001*	< 0.001*	< 0.001*	< 0.001*	< 0.001*	< 0.001*	< 0.001*	< 0.001*	< 0.001*
Sex													
Female													
mean	3.52	3.38	3.56	3.46	3.53	3.60	3.61	3.56	3.57	3.59	3.47	3.50	3.41
SD	1.21	1.19	1.33	1.13	1.34	1.32	1.31	1.39	1.28	1.33	1.18	1.24	1.04
Male													
mean	3.39	3.29	3.45	3.32	3.41	3.45	3.52	3.42	3.40	3.43	3.38	3.36	3.31
SD	1.24	1.20	1.36	1.17	1.37	1.36	1.36	1.46	1.32	1.35	1.21	1.29	1.07
*P* value	0.11	0.23	0.24	0.06	0.18	0.08	0.28	0.11	0.04*	0.07	0.22	0.08	0.15
Shift work													
Yes													
mean	2.16	2.12	2.1	2.24	2.08	2.14	2.2	2.4	2.15	2.14	2.24	2.13	2.34
SD	1.4	1.05	1.15	0.99	1.16	1.16	1.15	1.21	1.1	1.14	1.04	1.08	0.92
No													
mean	4.20	4.02	4.3	4.5	4.26	4.32	4.34	4.32	4.26	4.3	4.1	4.18	3.95
SD	0.5	0.58	0.6	0.58	0.63	0.59	0.63	0.66	0.6	0.65	0.62	0.58	0.57
*P* value	< 0.001*	< 0.001*	< 0.001*	< 0.001*	< 0.001*	< 0.001*	< 0.001*	< 0.001*	< 0.001*	< 0.001*	< 0.001*	< 0.001*	< 0.001*
Work unit													
Non-surgical													
mean	3.61	3.45	3.67	3.54	3.66	3.71	3.73	3.64	3.64	3.73	3.55	3.59	3.48
SD	1.15	1.15	1.27	1.05	1.30	1.24	1.29	1.36	1.22	1.27	1.13	1.20	0.99
Surgery													
mean	3.40	3.34	3.45	3.35	3.40	3.43	3.48	3.47	3.45	3.46	3.33	3.33	3.31
SD	1.28	1.30	1.39	1.21	1.43	1.37	1.28	1.49	1.34	1.38	1.25	1.34	1.14
Midwifery													
mean	3.53	3.47	3.64	3.43	3.51	3.57	3.53	3.61	3.55	3.59	3.51	3.54	3.44
SD	1.21	1.17	1.34	1.15	1.39	1.32	1.29	1.39	1.28	1.36	1.20	1.22	1.03
Pediatrics													
mean	3.48	3.22	3.41	3.44	3.50	3.60	3.67	3.60	3.50	3.46	3.60	3.48	3.33
SD	1.21	1.19	1.35	1.13	1.31	1.37	1.22	1.40	1.34	1.37	1.22	1.25	1.02
Psychiatry													
mean	3.83	3.72	3.91	3.73	3.68	4.15	3.95	3.92	3.91	3.83	3.65	3.90	3.71
SD	0.97	1.07	1.13	0.87	0.91	1.17	1.08	1.20	0.98	1.08	1.02	1.09	0.82
intensive care													
mean	3.41	3.29	3.45	3.35	3.41	3.47	3.51	3.44	3.45	3.45	3.39	3.40	3.33
SD	1.24	1.21	1.38	1.17	1.39	1.36	1.35	1.44	1.31	1.37	1.20	1.28	1.05
Emergency													
mean	3.50	3.33	3.56	3.45	3.56	3.57	3.61	3.56	3.52	3.59	3.45	3.44	3.41
SD	1.22	1.16	1.33	1.18	1.27	1.38	1.37	1.39	1.30	1.32	1.22	1.22	1.10
Lab													
mean	3.66	3.56	3.63	3.57	3.69	3.79	3.84	3.62	3.76	3.73	3.58	3.65	3.49
SD	1.13	1.11	1.19	1.12	1.21	1.27	1.35	1.30	1.32	1.21	1.12	1.18	0.90
Occupational medicine													
mean	3.61	3.42	3.67	3.44	3.66	3.71	3.81	3.67	3.72	3.69	3.65	3.53	3.47
SD	1.19	1.21	1.28	1.01	1.32	1.15	1.33	1.22	1.26	1.45	1.22	1.26	1.07
Operating room													
mean	3.40	3.17	3.50	3.17	3.33	3.83	3.50	3.50	3.50	3.39	3.44	3.33	3.21
SD	1.33	1.05	1.49	1.22	1.40	1.43	1.59	1.72	1.46	1.42	1.42	1.26	1.04
*P* value	0.55	0.62	0.57	0.61	0.57	0.23	0.45	0.72	0.56	0.41	0.70	0.46	0.64

* statistically significant result (*p* < 0.05)

## Abbreviations

AGFI: Adjusted goodness-of-fit index
AMOS: Analysis of a moment structures
ANOVA: Analysis of variance
CFI: Comparative fit index
CMIN/DF: Normed Chi-square
GFI: Goodness-of-fit index
HSOPSC: Hospital survey of patient safety culture
ICCs: Intraclass correlations
IFI: Incremental fit index
NFI: Normed fit index
PNFI: Parsimonious normed fit index
RMSEA: Root mean squared error of approximation
SPSS: Statistical package for the social sciences
